# A Case of Infective Endocarditis Due to Oral Streptococci After Perioperative Oral Function Management

**DOI:** 10.7759/cureus.20446

**Published:** 2021-12-15

**Authors:** Masanori Nashi, Shinsuke Yamamoto, Keigo Maeda, Naoki Taniike, Toshihiko Takenobu

**Affiliations:** 1 Oral and Maxillofacial Surgery, Kobe City Medical Center General Hospital, Kobe, JPN

**Keywords:** infective endocarditis, antimicrobial prophylaxis, oral streptoccci, invasive dental procedures, perioperative oral function management

## Abstract

Infective endocarditis is an extremely serious disease that can present with a variety of clinical manifestations, including infection of valves and endocardium, in patients with cardiac disease, and is associated with risk factors such as invasive dental procedures, caries, and periodontal disease. On the other hand, it has been shown that perioperative oral function management before various surgeries, such as those for malignant tumors, cardiovascular disease, and transplantation, may prevent or reduce postoperative complications. Close coordination between the dentist and cardiac surgeon is especially necessary before heart valve surgery because of the risk of severe complications. The number of perioperative oral management procedures being performed in community dental clinics is increasing. In the absence of clear guidelines, the physician-in-charge usually determines how to best perform oral management while considering the patient’s needs. We report a case of infective endocarditis occurring after perioperative oral management in a young patient with good oral hygiene. This case shows that standardization of the techniques and widespread dissemination of the guidelines are required. Patients should be counseled regarding the importance of maintaining oral hygiene from a young age.

This case report should act as a cautionary tale not only for hospital clinicians but also for community medical and dental practitioners, as the number of such patients is expected to increase in the future.

## Introduction

Infective endocarditis (IE) is a systemic septic disease characterized by the formation of vegetations containing bacterial communities on the valves, endocardium, and endothelium of large vessels. It presents with a variety of clinical symptoms. Oral *Streptococcus* and *Staphylococcus* species are the most common causative organisms. According to the Japanese guidelines, removal of infectious lesions and management of oral hygiene are important prophylactic procedures, and cooperation with dentists is essential for the prevention of IE associated with dental procedures [1].

Perioperative oral function management (POM) is one of the most important dental procedures for preventing the development of IE. There are many indications where POM may prevent or reduce postoperative complications [2-4]. In particular, close cooperation between the dentist and cardiac surgeon is necessary prior to heart valve surgery to minimize the risk factors for IE and the occurrence of surgical site infection. In recent years, the regional inter-disciplinary liaison has increased, which has led to an increase in the number of requests for POM received by general dental practitioners. However, there are no standardized criteria for performing POM, and it is up to the physician in charge to determine how to perform the procedure. Here, we report a case of IE caused by oral streptococci after POM.

## Case presentation

**Figure 1:**
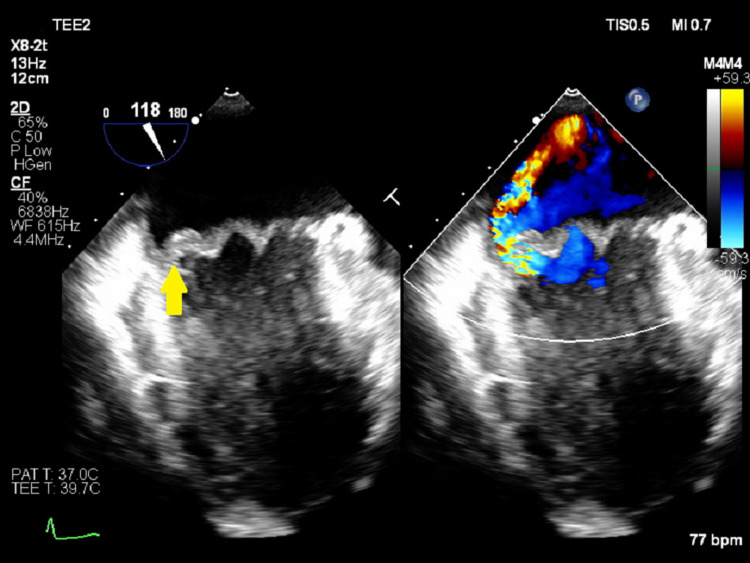
Transesophageal echocardiography findings revealed mitral regurgitation with vegetations (arrow), which had not been observed before.

A 30-year-old man with no prior medical history was referred to the Department of Cardiology at our hospital for further investigation and treatment of bicuspid aortic valve and aortic regurgitation with left ventricular enlargement. The patient was deemed a suitable candidate for surgery and was referred to the Department of Cardiovascular Surgery for further management. Informed consent was obtained for surgery and POM and the patient visited his family dentist for the procedure. Oral care, involving invasive dental procedures, was performed without prior administration of antimicrobials. The patient developed a low-grade fever a few days after POM, without any specific symptoms, for which he did not seek medical help. The patient underwent autologous blood transfusion eight days after POM because he had not developed any other symptoms except for the low-grade fever. At that time, transthoracic echocardiography revealed no evidence of decreased cardiac function or presence of vegetations. He was admitted for surgery 10 days after POM. He did not have a fever, but blood tests showed acute inflammation; therefore, the surgery was postponed and a systemic examination was performed. A whole-body computed tomography scan showed no obvious abnormalities; therefore, treatment with antimicrobial agents was started. On the fourth day of hospitalization, *Streptococcus mutans* was detected in all blood cultures taken at the time of hospitalization, and transesophageal echocardiography revealed mitral regurgitation with vegetations, which had not been observed before (Figure 1).

These findings led to a definitive diagnosis of IE according to the modified Duke criteria [5]. Therefore, he was referred to our department for the investigation of the cause and evaluation of the oral condition.

**Figure 2:**
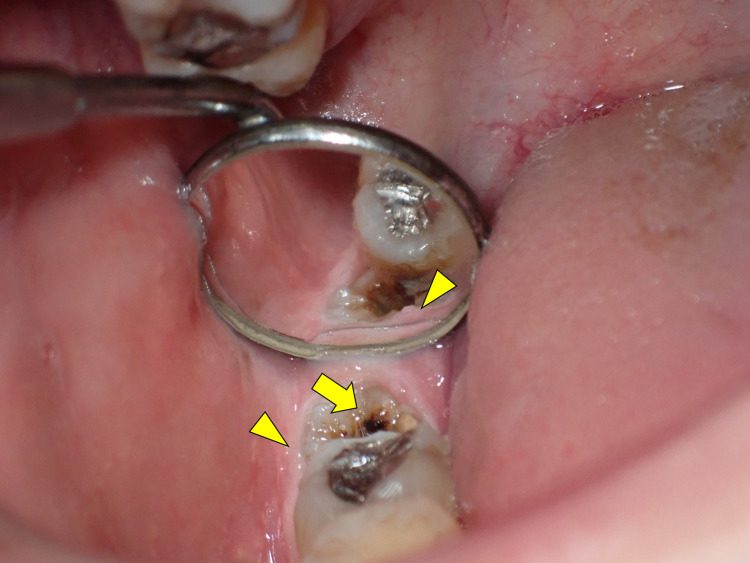
Intraoral photograph showing the stained occlusal surface of the third molar of the right mandible (arrow), and mild inflammatory findings of the marginal gingiva were observed (arrowhead).

The patient had good oral hygiene, and there was no evidence of deep caries and tooth mobility that could cause hematogenous infection. The occlusal surface of the third molar of the right mandible was stained, but the patient did not report any accompanying symptoms (Figure 2).

**Figure 3:**
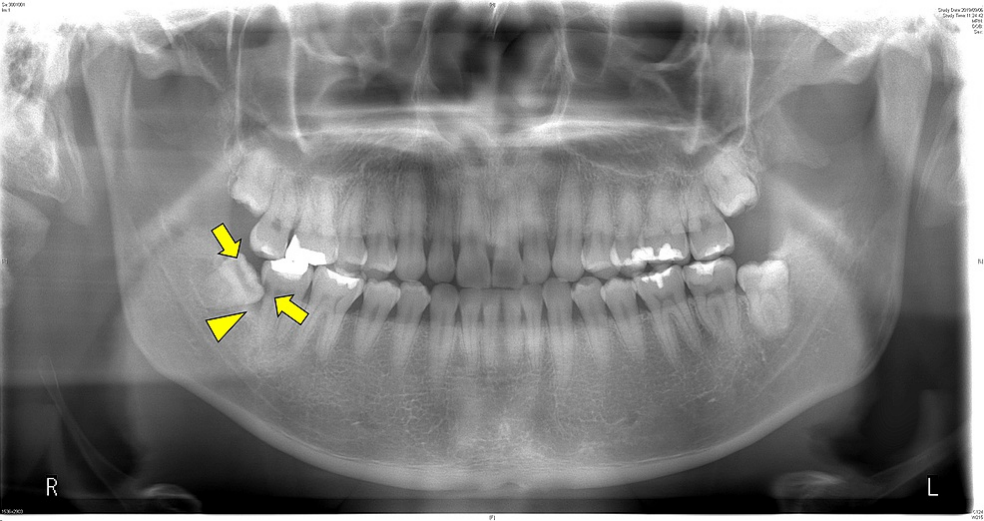
Panoramic radiograph showing a radiolucent lesion distal to the proximal surface of the second molar of the right mandible (yellow arrow) and pericoronitis around the periapical area of the third molar (arrowhead).

Panoramic radiograph revealed a radiolucent lesion distal to the proximal surface of the second molar of the right mandible and pericoronitis around the periapical area of the third molar (Figure 3).

No other obvious abnormalities were observed. The clinical diagnosis was IE caused by a dental infection. After consultation with the attending physician, it was decided that the suspected source of infection should be immediately treated, and the patient underwent extraction of the third molar and treatment for caries of the second molar of the right mandible.

The postoperative course was uneventful, and follow-up was completed after good healing was confirmed. The patient then underwent valve replacement and valvuloplasty and the postoperative course was good.

## Discussion

POM is being widely recognized as a necessary procedure during the perioperative period to prevent oral cavity-related postoperative complications, and reduce the length of hospital stay, and is now being performed in many facilities throughout Japan [2-4]. However, if a physician does not update his/her clinical knowledge regarding this procedure and learn how to modify it according to the patient’s needs, he or she may inadvertently increase the risk before surgery, as in this case.

This case has two important implications for clinicians. First, continuous medical education for dentists, uniform criteria for POM, and widespread dissemination of the guidelines are needed. There are no criteria for proper selection and treatment of the patients, and all decisions regarding POM are made by the dentist-in-charge. Consequently, there is a possibility that lesions that should be treated may be overlooked. There are some recommendations regarding antimicrobial prophylaxis before dental procedures, but a consensus has not been reached [1,6-8].

According to the Japanese guidelines [1], the patient should be administered antimicrobial prophylaxis after consultation with a doctor. However, POM was performed without antimicrobial prophylaxis. The dentists should discuss whether the patient needs antimicrobial prophylaxis with the physicians to determine how to handle the situation.

Second, regular guidance regarding the maintenance of oral hygiene is important, even for healthy young people. In the present case, there were two possible routes of infection, namely, invasive dental procedures performed during POM, and chronic dental caries resulting in bacteremia during eating and brushing. Nakamura et al. reported that tooth extraction should be performed up to 14 days before surgery because of the increased risk of IE during the first 14 days after the onset of bacteremia; no similar report was found for scaling and tooth cleaning [9]. The timing and details of the procedures performed during POM should be verified by accumulating more data in the future. Although asymptomatic non-progressive caries is not clearly defined as a risk factor for IE, the presence of chronic caries may lead to severe bacteremia and IE, as in this case. Oral bacteria are generally susceptible to antimicrobial drugs, but dental biofilm is difficult to eradicate with antimicrobial therapy. The presence of biofilm in semi-erupted wisdom teeth, which are prone to localized caries and periodontal disease, may be a risk factor for IE. Dentists must keep this in mind when performing POM.

Tomas et al. reported that the number of patients with IE originating from oral diseases, who underwent invasive dental procedures and those who did not, was the same, suggesting the need for constant attention to the oral environment in patients at high risk for developing IE, even in the absence of clinical symptoms [10]. It is known that transient bacteremia occurs even with daily brushing and eating [1], but it is considered to be less risky than invasive dental procedures. However, it has been pointed out that the cumulative time of bloodstream infection may pose a risk equal to or greater than that of invasive dental procedures [7]. Therefore, all patients should maintain good oral hygiene and the clinicians should counsel their patients more about this issue. 

## Conclusions

IE caused by oral streptococci is known to follow a subacute course and is believed to be less severe. However, effort should be made to ensure its prevention due to the possibility of serious complications associated with IE.

This case suggests the need to create uniform guidelines for POM and to standardize dental procedures. We hope that this will be an opportunity for clinicians to learn more about the importance of maintaining good oral hygiene from a young age and counseling their patients regarding this.
